# Supplementary material for: Neutrophilic inflammation promotes SARS-CoV-2 infectivity and augments the inflammatory responses in airway epithelial cells
Source: Front Immunol. 2023 Mar 16;14:1112870. doi: 10.3389/fimmu.2023.1112870 (PMC10061003; doi:10.3389/fimmu.2023.1112870)
Supplement: Supplementary file 1 [file DataSheet_1.pdf]

Supplemental Information:

Related to manuscript "Neutrophilic inflammation promotes SARS-CoV-2 infectivity and augments the inflammatory responses in airway epithelial cells".

Supplemental Methods

***In vitro* airway differentiation: Spheroids**

For differentiated spheroids, transwells were pre-treated with 25% (v/v) of Matrigel in Pneumacult Ex+ (Stem Cell Technologies) for at least 1 hour at 37°C. For spheroid formation, a single cell suspension of HBECs was generated using with Accutase. Single cells were resuspended in Matrigel™ 5% in Pneumacult Ex+. The cell suspension was loaded onto the pre-established gel-bed and Pneumacult Ex+ media added to the lower chamber. After 3 days the media was exchanged for Pneumacult ALI maintenance media (Stem Cell Technologies) and changed every 2~3 days. Spheroids were fixed with 4% PFA in PBS at 5, 10, 14 and 20 days after the culture. For immunostaining, spheroids, attached at the bottom of the transwells, were pre-treated with 0.1% of Triton X-100 in PBS (hereinafter 0.1% PBT) for 10 min for permeabilization. The cells were incubated with primary antibody for 1hr 30min at room temperature and washed with 0.1% PBT. Secondary antibodies were incubated for 45 min at room temperature and washed with 0.1% PBT. Nuclei were stained with DAPI. The spheroids were mounted with Fluoromount G (#0100-01; SouthernBiotech). The spheroid images were taken with confocal microscope (Zeiss, LSM710) using a Plan Apochromat 63x/1.4 NA oil-immersion objective, ~10 μm of thickness with 0.4μm of z-stack interval and processed with ZEN software (Zeiss) and ImageJ software.

Single Cell RNA sequencing and Bioinformatic Analysis

Gene expression counts (GEO accession number: GSE150674, [1]) from both the fresh isolates and ALI cultures were processed using functions in the R package Seurat (version 4.0.2) [2]. For each sample, low quality cells and potential cell doublets were removed by filtering cells based on the number of genes detected and the fraction of reads mapping to the mitochondrial genome. Cells with less than 200 genes detected or greater than 4,900 – 7,000 genes detected (depending on the sample) were removed as well as cells with greater than 10% of mitochondrial reads. The filtered gene counts were then integrated into a unified data set using the

SCTransform [3] based integration workflow implemented in Seurat. The first 30 PCs were used as the number of dimensions for the FindNeighbors and FindClusters functions. To find the optimal clustering resolution value we explored a range of resolution values from 0 to 2 by increments of 0.2. We then visualized the range of cluster resolutions using the R package clustree (version 0.4.3) [4]. A final resolution of 0.4 was selected and used for downstream analyses. Clusters were visualized in two-dimensional space using the RunUMAP function in Seurat. Cluster specific cell types were annotated manually by examining the expression of canonical marker genes within a given cluster and clusters with shared marker gene expression patterns were collapsed. The fresh isolates and ALI couture data sets were analyzed separately.

Supplemental Results

Additions to the main text references to the supplemental data

Expression of ACE2 and TMPRSS2 in human lung and *in vitro* human lung models. To validate the expression patterns, we re-analyzed published single cell sequencing datasets (GEO accession number: GSE150674) on proximal human lung tissues and found that ACE transcripts were low in abundance and detectible levels of both ACE2 and TMPRSS2 were found in ciliated cells, SMG cells, basal cells, and secretory cells (**supplementary figure S2a-b**). At the protein level expression of ACE2 was observed most notably in ciliated columnar epithelial cells (**supplementary figure S2c**) and in submucosal glands (**supplementary figure S2e**). TMPRSS2 was found to be more ubiquitous in its expression and found throughout the tissue (**supplementary figure S2d&f**) inclusive of strong expression in the ciliated epithelium (**supplementary figure S2d**) and in submucosal glands (**supplementary figure S2f**).

Expression of ACE2 in infiltrating neutrophils in human lung and *in vitro* human lung models. The presence of a very high ACE2 expressing cell (**supplementary figure S5a)** with a polynuclear migratory phenotype was noted to be located in blood vessels, tissue parenchyma and infiltrating the epithelium. This cell type was substantially more prevalent in CF tissues (**supplementary figure S5b)** compared to patients with no underlying chronic respiratory disease (**supplementary figure S5a)** and co-localized with Neutrophil Elastase (NE) **(supplementary figure S5c)** and CD15 (**supplementary figure S5d**), confirming the cells as neutrophils.

To confirm neutrophil expression of ACE2 CD15+ peripheral blood polynucleocytes were isolated and co-stained with NE and ACE2 (**supplementary figure S5e**).

Supplemental Tables

Supplemental table S1: Demographic information for primary human blood donors for neutrophil isolation.

Identifier	Disease Sate	Age	Gender
BD001	No underlying disease	40	Female
BD002	No underlying disease	33	Male
BD003	No underlying disease	23	Female
BD004	No underlying disease	28	Female

Supplemental table S2: Demographic information for lung tissue donors used in this project.

Identifier	Disease	Age	Gender	BMI	Co-morbidities	Source
Au20-39	COVID-19	39	Male	53.2 Kg/m^2	None reported	UVM
Au20-48	COVID-19	72	Male	29.1 Kg/m^2	GI Reflux, Prostate CA s/p radiation, hyperlipidemia, former smoker, hypertension	UVM
004-H	No-underlying disease	UK	UK	UK	UK	UI
016-CF	Cystic Fibrosis (ΔF508/I633K)	54	Female	N/A	Bronchiectasis	UI
018-H	No-underlying disease	UK	UK	UK	UK	UI
019-CF	Cystic Fibrosis (ΔF508/G551D)	24	Female	N/A	None reported	UI

UK, unknown, UI, University of Iowa, UVM, University of Vermont

Supplemental table S3: Demographic information for primary human bronchial epithelial cell donors and pairings with blood donors for ALI co-cultures.

Identifier	Disease	Age	Gender	BMI	Co-morbidities	Source	Blood Donor Pairing
044-H	None known	73	Female	24.8 Kg/m^2	Seizures, Epilepsy, early signs of dementia	IIAM	BD001
033-H	None known	UK	UK	UK	UK	UI	BD002
014-H	None known	24	Male	UK	UK	UNC-Chapel Hill	BD002
032-H	None known	UK	UK	UK	UK	UI	BD003
015-H	None known	UK	UK	UK	UK	UI	BD003
017-H	None known	35	Female	UK	UK	UNC-Chapel Hill	BD004

UK, unknown, UI, University of Iowa, UNC-Chapel Hill, University of North Carolina-Chapel Hill

Supplemental table S4: Data table complementing Figures 1b, 1c, 1d and 3e. Data is expressed as mean±S.E.M.

Figure 1b		Neutrophils	-	+	-	+
		SV2	-	-	+	+
	Cytokine					
	IFNγ	pg/ml	112.9±14.8	716.2±30.06	234.9±13.46	2419±165.9
	IL1-β	pg/ml	283.7±48.74	380.2±51.76	360.9±27.47	610.9±95.29
	IL-4	pg/ml	56.2±13.4	55.58±12.31	99.98±19.26	122.9±23.4
	IL-6	pg/ml	804.1±464.7	1455±497.1	1488±778.3	8629±1161
	IL-8	pg/ml	0±0	0±0	0±0	0±0
	IL-10	pg/ml	106±22.86	289.8±38.86	169.6±27.38	392.9±56.21
	TNFα	pg/ml	183±40.06	398±109.5	314.3±51.52	602.4±183.6

Figure 1c		Neutrophils	-	+	-	+
		SV2	-	-	+	+
	Cytokine					
	IFNγ	pg/ml	36.8±6.823	129.6±	49.72±	129.6±
	IL1-β	pg/ml	51.92±9.39	289.2±9.755	54.32±8.725	310.8±12.62
	IL-4	pg/ml	50.57±4.721	111.5±4.611	50.63±7.727	114.9±5.889
	IL-6	pg/ml	206.6±17.84	761.9±120.7	158.2±23.77	1115±200.2
	IL-8	pg/ml	6180±2248	0±0	52996±13724	0±0
	IL-10	pg/ml	74.42±15.36	253.9±17.4	142.4±12.82	397.3±21.34
	TNFα	pg/ml	82.43±14.83	274±57.8	145.1±28.3	459.1±48.02

Figure 1d		IFNγ	-	+	-	+
		SV2	-	-	+	+
	Cytokine					
	IFNγ	pg/ml	66.29±20.38	1060±109.4	41415±6957	18089±1747
	IL1-β	pg/ml	0.04578±0.003297	152.3±15.5	0.1273±0.02164	614.2±45.14
	IL-4	pg/ml	0.03828±0.003686	1.109±0.08929	0.2378±0.03885	3.11±0.1308
	IL-6	pg/ml	0.2277±0.02615	579.5±98.12	0.6101±0.1096	2395±32.93
	IL-8	pg/ml	8.693±1.465	2315±123.9	9.438±1.405	1489±116.1
	IL-10	pg/ml	0.08593±0.005330	3.421±1.447	0.2358±0.03930	6.003±1.087
	TNFα	pg/ml	0.0131±0.005618	161.1±18.80	0.277±0.05815	2587±162.9

Figure 3e		Neutrophils	-	+	-	+
		SV2	-	-	+	+
	Infected Cells					
	KRT-	%	1.656±1.524	0±0	15.22±3.130	33.2±8.304
	KRT+	%	0.1449±0.1449	0±0	11.87±2.570	51.1±7.544
	Total	%	0.9158±0.8489	0±0	12.45±1.805	40.65±7.724

Figure 3f		Neutrophils	-	+	-	+

		SV2	-	-	+	+
	**KRT+ Thickness**	**μm**	16±0.3017	14.55±0.2610	14.85±0.3343	20.15±0.5834
	**Total Thickness**	**μm**	38.5±0.3021	33.94±0.1878	33.35±0.3738	42.32±0.7685
	**Total Cells**	**Count**	123.2±6.187	135.7±4.840	116.9±4.687	137.5±6.095

Supplemental table S5: Key resources table.

REAGENT or RESOURCE	SOURCE	IDENTIFIER
Antibodies		
Chicken Polyclonal anti Cytokeratin 5 (KRT5)	Biolegend	Cat# 905901; RRID:AB_2565054
Rat monoclonal anti Angiotensin Converting Enzyme – 2 (ACE2)	Biolegend	Cat# A20069I; RRID:AB_2860959
Rabbit polyclonal anti Angiotensin Converting Enzyme – 2 (ACE2)	Abcam	Cat# ab15348; RRID:AB_301861
Goat polyclonal anti Epithelial Cell Adhesion Molecule (EpCAM)	R&D Systems	Cat# AF960; AB_355745
Mouse monoclonal anti α-Tubulin (ATUB)	Abcam	Cat# ab24610; RRID:AB_448182
Rabbit monoclonal anti Transmembrane Protease Serine 2 (TMPRSS2)	Abcam	Cat# ab109131 RRID:AB_10863728
Rabbit monoclonal anti Neutrophil Elastase (NE)	Thermo Fisher Scientific	Cat# MA5-32548; RRID:AB_2809825
Mouse monoclonal anti Cluster of Differentiation 15 (CD15)	Thermo Fisher Scientific	Cat# MA5-17042; RRID:AB_2538514
Mouse monoclonal anti Cluster of Differentiation 15 (CD15) – PE Conjugated	Biolegend	Cat# 323005 RRID:AB_756011
Rabbit polyclonal anti SARS-CoV-2 spike glycoprotein	Abcam	CAT# ab272504 RRID: AB_2847845
RNAscope Probe - V-nCoV2019-S	ACDBio	Cat# 848561
Bacterial and Virus Strains		
SARS-CoV-2 Isolate USA-WA1/2020	BEI Resources	Cat# NR-52281
Biological Samples		
AU20-039 Post-mortem COVID human lung tissue	University of Vermont	N/A
AU20-043 Post-mortem COVID human lung tissue	University of Vermont	N/A
004-H Post-mortem no underlying respiratory disease lung tissue	Laboratory of Amy Ryan	N/A
016-CF Explanted transplant Cystic Fibrosis Human lung tissue	Laboratory of Amy Ryan	N/A
018-H Post-mortem no underlying respiratory disease lung tissue	Laboratory of Amy Ryan	N/A
019-CF Explanted transplant Cystic Fibrosis Human lung tissue	Laboratory of Amy Ryan	N/A
Chemicals, Peptides, and Recombinant Proteins		
Triton-X	Sigma Aldrich	Cat# 9002-93-1
Paraformaldehyde	Thermo Fisher Scientific	Cat# 28908
Human recombinant Tumor Necrosis Factor-Alpha Research Grade (TNFα)	Miltenyi Biotec	Cat# 130-094-015
Human Recombinant Interleukin 6 (IL6)	Miltenyi Biotec	Cat# 130-095-365
Human Recombinant Interleukin 1-beta (IL1β)	Miltenyi Biotec	Cat# 130-093-895
Human Recombinant Interferon-gamma (IFNγ)	PeproTech	Cat# 300-02-100ug
Flash Phalloidin Red 594	Biolegend	Cat# 424203
Cytochalasin D	VWR	Cat# C8273-1MG
Trizol™	Thermo Fisher Scientific	Cat# 15596026
Pneumacult-EX Plus Medium	Stem Cell Technologies	Cat# 05040
Pneumacult-ALI Medium	Stem Cell Technologies	Cat# 05001
Hanks balanced Salt Solution	Invitrogen	Cat# 14025076

B-ALI Growth Medium	Lonza	Cat# 00193516
Phosphate Buffered Saline	Invitrogen	Cat# 14190-250
Foetal Bovine Serum	Fisher Scientific	Cat# 16-141-061
Ethylenediaminetetraacetic acid	Thermo Fisher Scientific	Cat# AM9261
TruStain Fc receptor blocker	Biolegend	Cat# 422302
Airway Epithelial Growth Medium	Promocell	Cat# C-21060
Accutase	Stem Cell Technologies	Cat# 07920
Dulbecco's Modified Eagles Medium	Thermo Fisher Scientific	Cat# 11995073
Puromycin	Thermo Fisher Scientific	Cat# A1113802
OptiMEM	Thermo Fisher Scientific	Cat# 31985062
**Critical Commercial Assays**		
MSD Cytokine Assay V-plex Viral Panel 1 Human Kit	MSD	Cat# K15345D-1
MSD SARS-CoV-2 Spike S-PLEX	MSD	Cat#K150ADJS
QuantiTect Virus Kit	Qiagen	Cat# 211011
Direct-zol RNA Microprep kit	Zymo	Cat# R2061
SARS-CoV-CDC RUO primers and probes	Integrated DNA Technologies	Cat# 1000673
EasySep direct neutrophil isolation kit	Stem Cell Technologies	Cat# 19666
Hematoxylin	VWR	Cat# 10143-612
Eosin	VWR	Cat# 10143-132
Bovine Serum Albumin	RPI	Cat# A30075-100.0X
Normal Donkey Serum	Jackson Immuno Research	Cat# 017-000-121
Paraformaldehyde	Thermo Fisher Scientific	Cat# 28908
Fluoromount-G	SouthernBiotech	Cat# 0100-01
4′,6-diamidino-2-phenylindole	Thermo Fisher Scientific	Cat# D1306
Experimental Models: Cell Lines		
Human Basal Epithelial Cells (HBECs) 032H	Laboratory of Amy Ryan	N/A
Human Basal Epithelial Cells (HBECs) 015H	Laboratory of Amy Ryan	N/A
Human Basal Epithelial Cells (HBECs) 017H	Laboratory of Amy Ryan	N/A
Human Basal Epithelial Cells (HBECs) 044H	Laboratory of Amy Ryan	N/A
Human Basal Epithelial Cells (HBECs) 033H	Laboratory of Amy Ryan	N/A
Human Basal Epithelial Cells (HBECs) 014H	Laboratory of Amy Ryan	N/A
Human CD15+ Neutrophils BD001	Laboratory of Amy Ryan	N/A
Human CD15+ Neutrophils BD002	Laboratory of Amy Ryan	N/A
Human CD15+ Neutrophils BD003	Laboratory of Amy Ryan	N/A
Human CD15+ Neutrophils BD004	Laboratory of Amy Ryan	N/A
VeroE6-hACE2	Laboratory of Dr. Jae Jung, USC	

Oligonucleotides		
2019-nCoV_N1-F 2019-nCoV_N1 Forward Primer GAC CCC AAA ATC AGC GAA AT	Integrated DNA Technologies	Cat# 1000673
2019-nCoV_N1-R 2019-nCoV_N1 Reverse Primer TCT GGT TAC TGC CAG TTG AAT CTG	Integrated DNA Technologies	Cat# 1000673
2019-nCoV_N1-P 2019-nCoV_N1 Probe FAM-ACC CCG CAT TAC GTT TGG TGG ACC-BHQ1 FAM, BHQ-1 125nM	Integrated DNA Technologies	Cat# 1000673
RP-F RNAse P Forward Primer AGA TTT GGA CCT GCG AGC G	Integrated DNA Technologies	Cat# 1000673
RP-R RNAse P Reverse Primer GAG CGG CTG TCT CCA CAA GT	Integrated DNA Technologies	Cat# 1000673
RP-P RNAse P Probe: FAM – TTC TGA CCT GAA GGC TCT GCG CG – BHQ-1	Integrated DNA Technologies	Cat# 1000673
Software and Algorithms		
Image J	Fiji – NIH	
Graph Pad Prism	GraphPad	
BD *FACSDiva*™	BD Biosciences	
FlowJo	BD Biosciences	
R Package Seurat	R Foundation	
R Package Clustree	R Foundation	
Leica LAS X Navigator	Leica	
Discovery Workbench	MSD	
ZEN lite	Zeiss	

Supplemental figures and figure legends

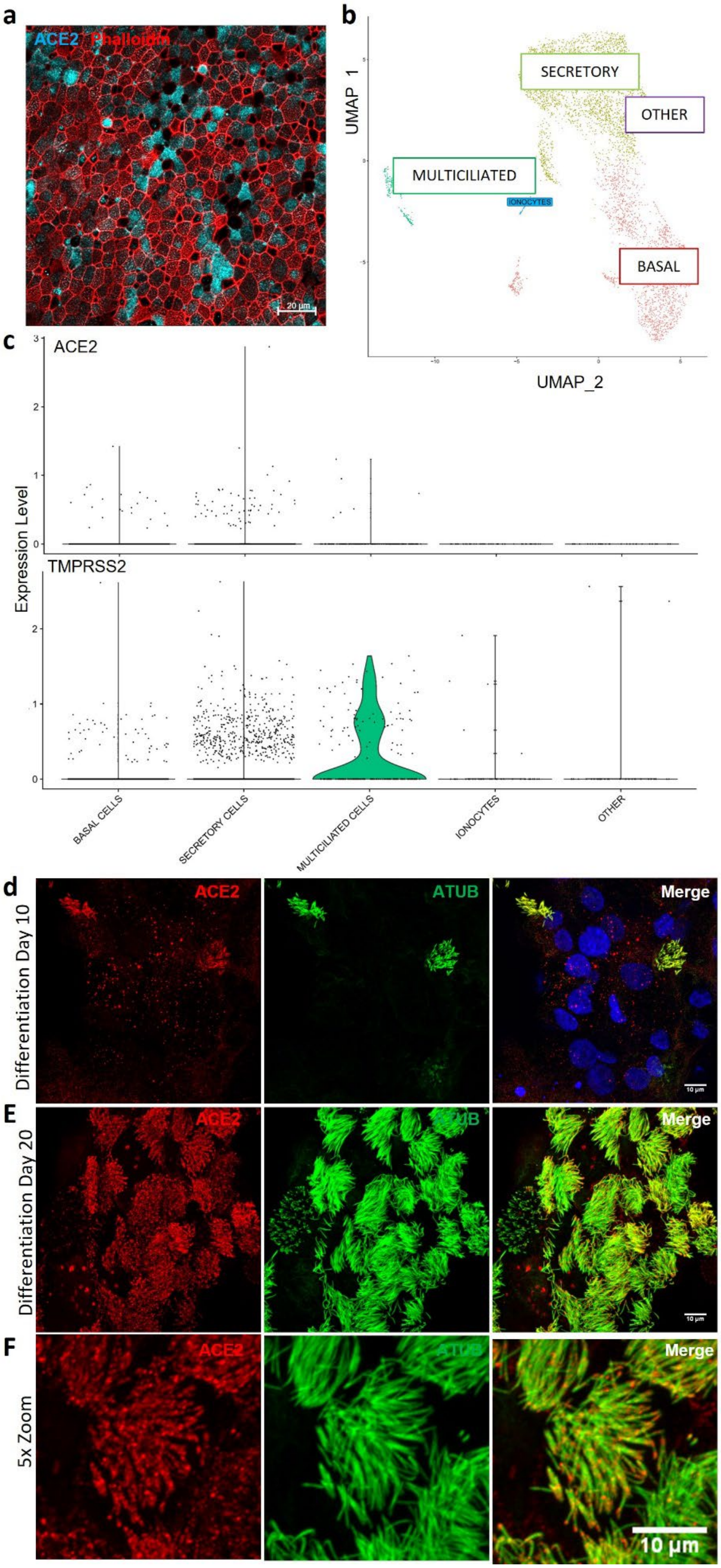

Supplemental figure S1, related to figure 1: ACE2 expression in *in vitro* ciliated cell basal bodies and axonemes. a) Representative immunofluorescent image of mature airway epithelium differentiated at the Air-Liquid-Interface probed for ACE2 (Cyan) and co-stained for tight junctions with phalloidin (Red). b) Single cell sequencing cluster analysis visualized by Uniform Manifold Approximation and Projection (UMAP) of cells differentiated at the Air-Liquid-Interface. c) Expression analysis of ACE2 (top panel) and TMPRSS2 (bottom panel) of differential clusters identified UMAP analysis of single cells isolated from differentiated cultures at the Air-Liquid-Interface. D-f) representative immunofluorescent images of tracheospheres probed for ACE2 (red) and acetylated-alpha tubulin (Green) depicting an increase in expression from day 10 differentiation (d) to day 20 differentiation (e) with co-localization of ACE2 at the basal bodies of the cilia (f). Images have nuclei counterstained with DAPI and scale bars are 20 μm in A or 10 μm in d-f.

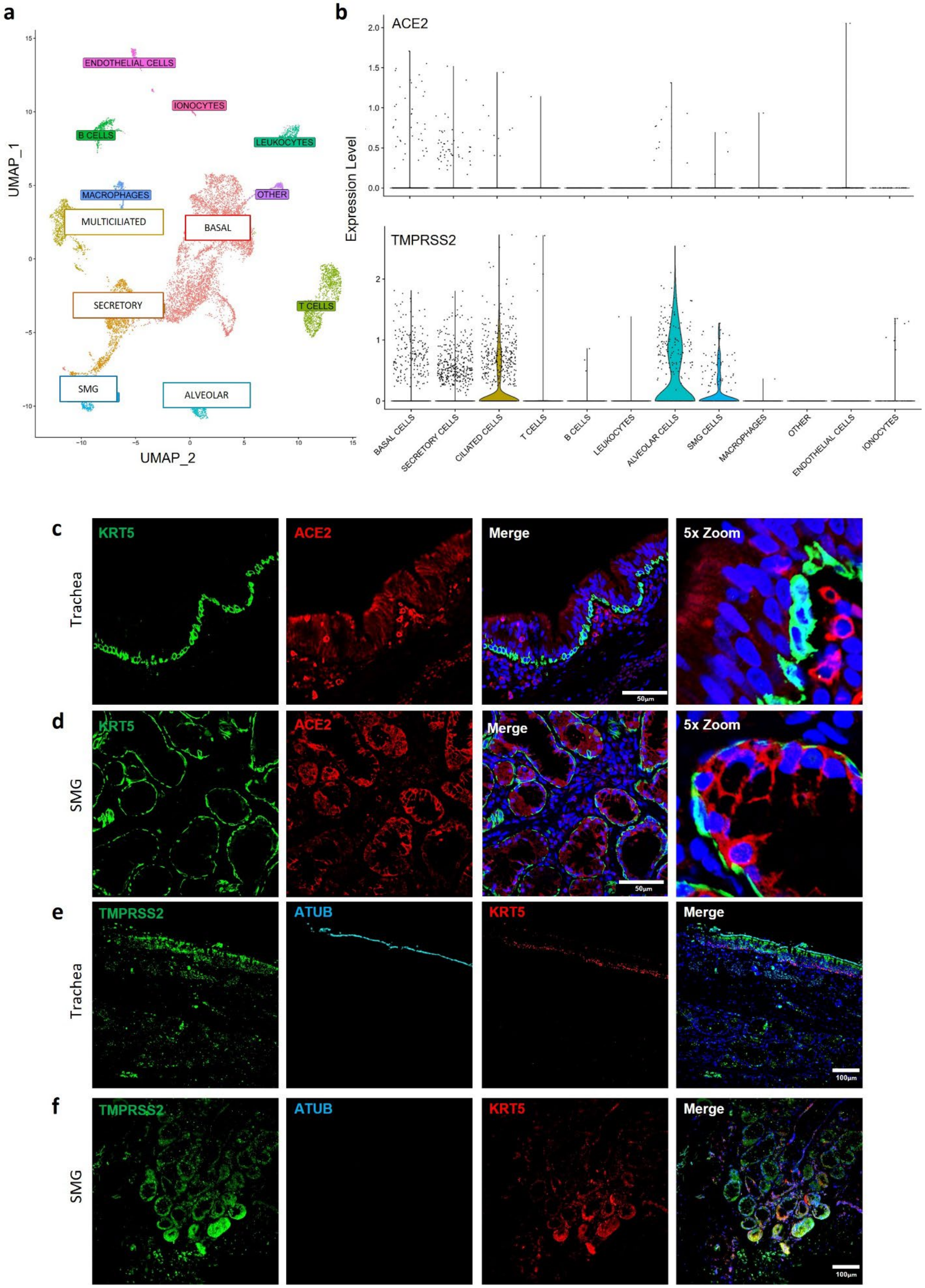

Supplemental figure S2, related to figure 1: ACE2 and TMPRSS2 expression profiles across human lung upper airways: a) Single cell sequencing cluster analysis visualized by Uniform Manifold Approximation and

Projection (UMAP) of cells isolated from primary human lung tissue. b) Expression analysis of ACE2 (top panel) and TMPRSS2 (bottom panel) of differential clusters identified UMAP analysis of single cells isolated from human lung tissue. Representative immunofluorescent (IF) images of: c) primary human lung trachea surface airway epithelium and d) submucosal glands (SMG) stained for cytokeratin 5 (KRT5, green) and ACE2 (red); e) primary human lung trachea surface airway epithelium and f) submucosal glands (SMG) stained for TMPRSS2 (green), acetylated α tubulin (ATUB, cyan) and KRT5 (red). All images have nuclei counterstained with DAPI (blue) and scale bars represent 50 μm in C and D and 100 μm in e and f. All images are representative of 3 of 3 independent regions per donor at least 2 independent donors

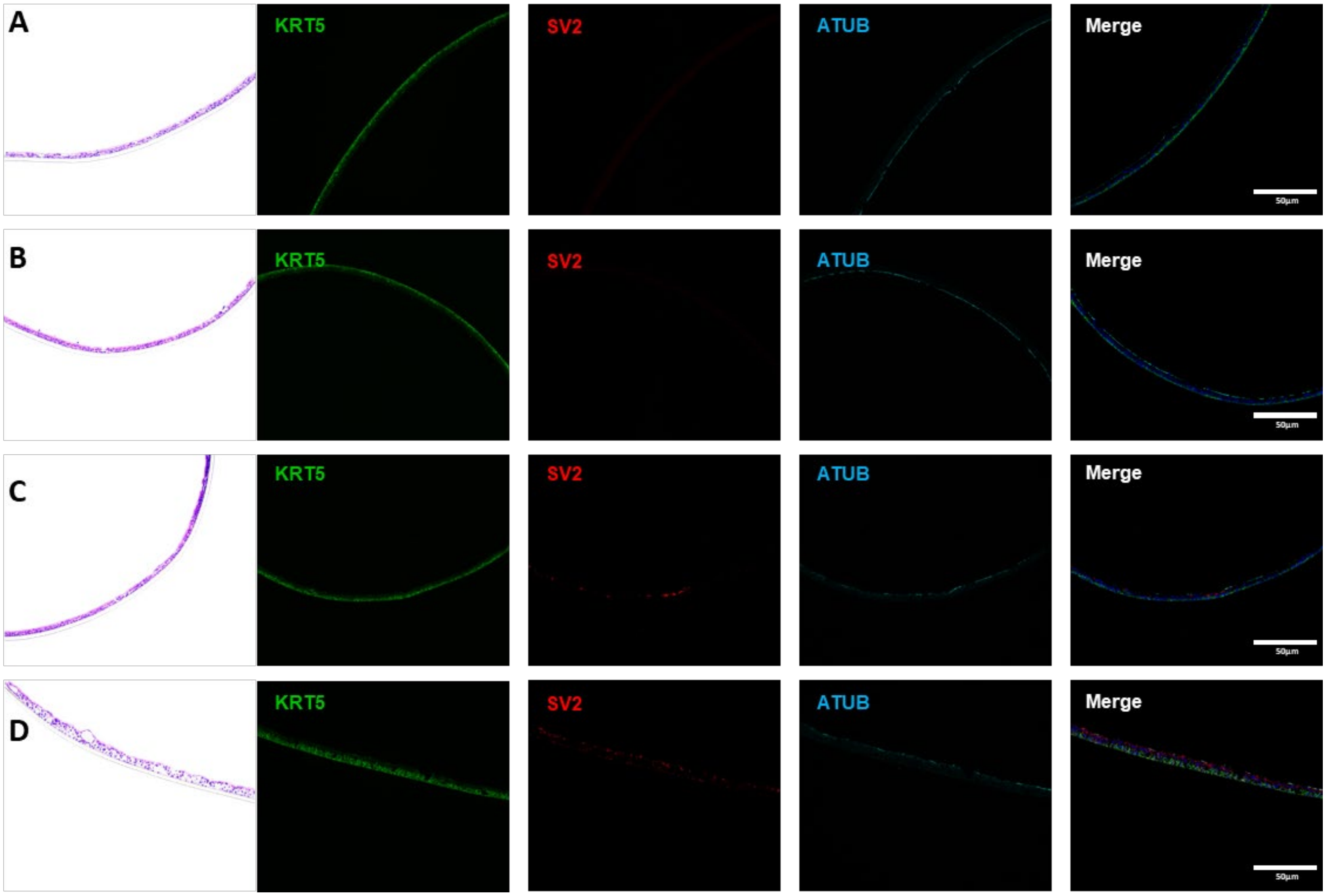

Supplemental figure S3, related to figure 3: **A pre-existing pro-inflammatory environment increases SARS-CoV-2 infection of airway basal stem cells**. a-d) representative hematoxylin and eosin (H&E) staining and immunofluorescent images of cross section culture models probed for KRT5 (green) SARS-CoV-2 (red) and alpha-tubulin (cyan) at 10x zoom. a) uninfected monocultured epithelial cells. b) uninfected epithelial cell – neutrophil co-culture. c) SARS-CoV-2 infected epithelial cell monoculture. d) SARS-CoV-2 infected epithelial cell – neutrophil co-culture. All IF images have nuclei counterstained with DAPI (blue) and scale bars represent 50μm. All images are representative of 3 independent experimental repeats of 3 neutrophil and 3 epithelial random donor pairings.

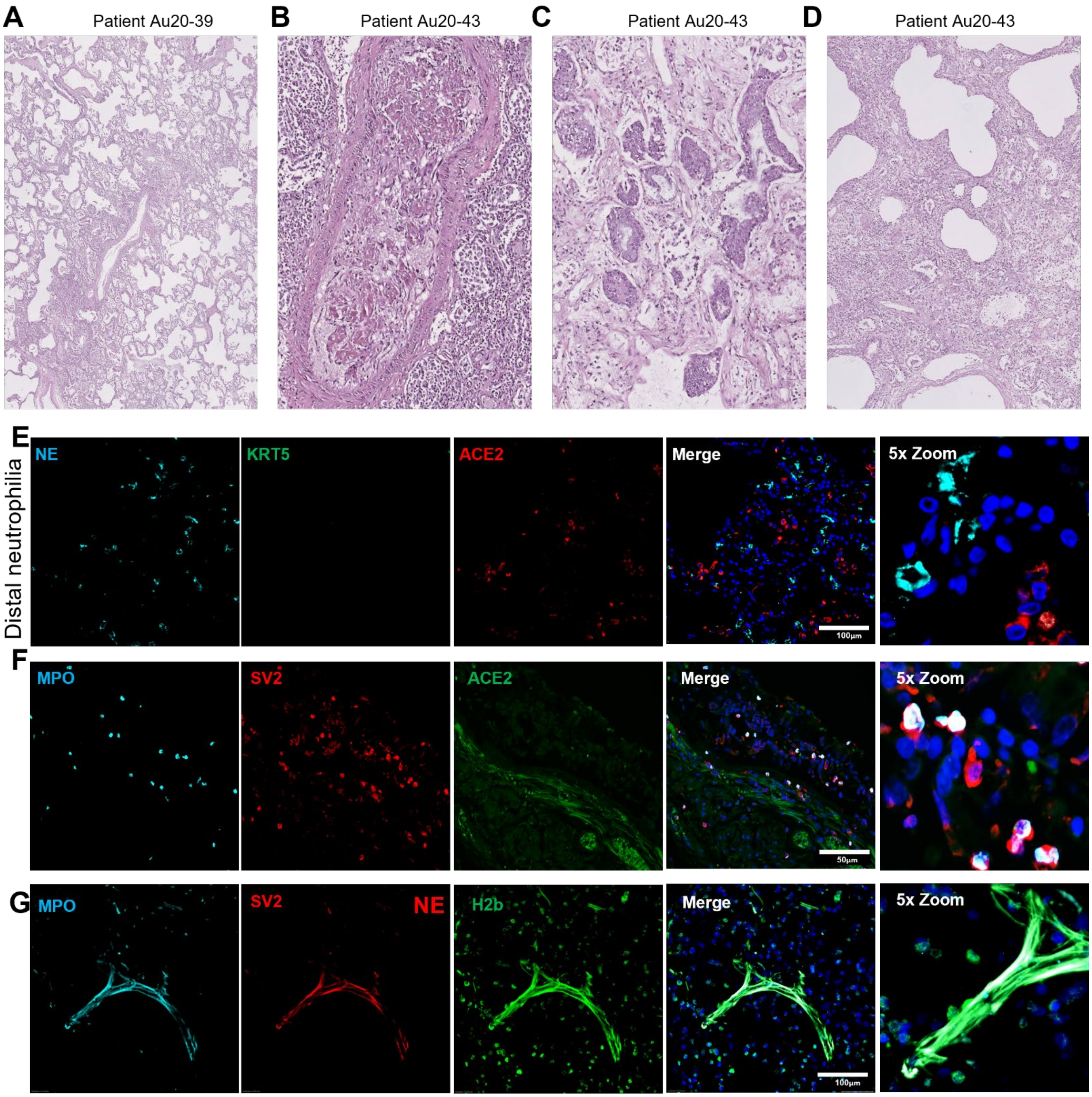

Supplemental figure S4, related to figure 4: Neutrophil associated tissue pathology in post-mortem COVID19 and underlying respiratory disease in human lung airways. A-d) Representative H&E staining of postmortem COVID-19 patient tissues (a) patchy organizing pneumonia centered on an artery and adjacent airway (H&E; original magnification 20x); b) Artery involved by an organizing thrombus. The adjacent alveolar sacs are filled with inflammatory cells including numerous foamy histiocytes (H&E; original magnification 100x).

c) Area of peribronchiolar metaplasia with early squamous metaplasia. Peribronchiolar metaplasia is a common finding and may have existed prior to the COVID infection, but the squamous metaplasia appears to be in early phase and is likely a component of the COVID-induced tissue injury (H&E; original magnification 100x). d) Rounded airspaces are within the interstitium and lined by organized inflammatory cells and fibrin, consistent with barotrauma from mechanical ventilation (H&E; original magnification 40x). e) Representative IF image probed for NE (cyan), SARS-CoV-2 (red) and ACE2 (green) f) Representative image of Myeloperoxidase (MPO) activity centered around SARS-CoV-2 infection, probed for MPO (cyan), SARS-CoV-2 (red) and ACE2 (green). g) Evidence for Netosis in post-mortem COVID-19 tissue, probed for MPO (cyan), SARS-CoV-2 (red) and H2b (green). All IF images have nuclei counterstained with DAPI (blue) and scale bars represent 50 or 100μm. All images are representative of 3 independent regions per donor at least 2 independent donors.

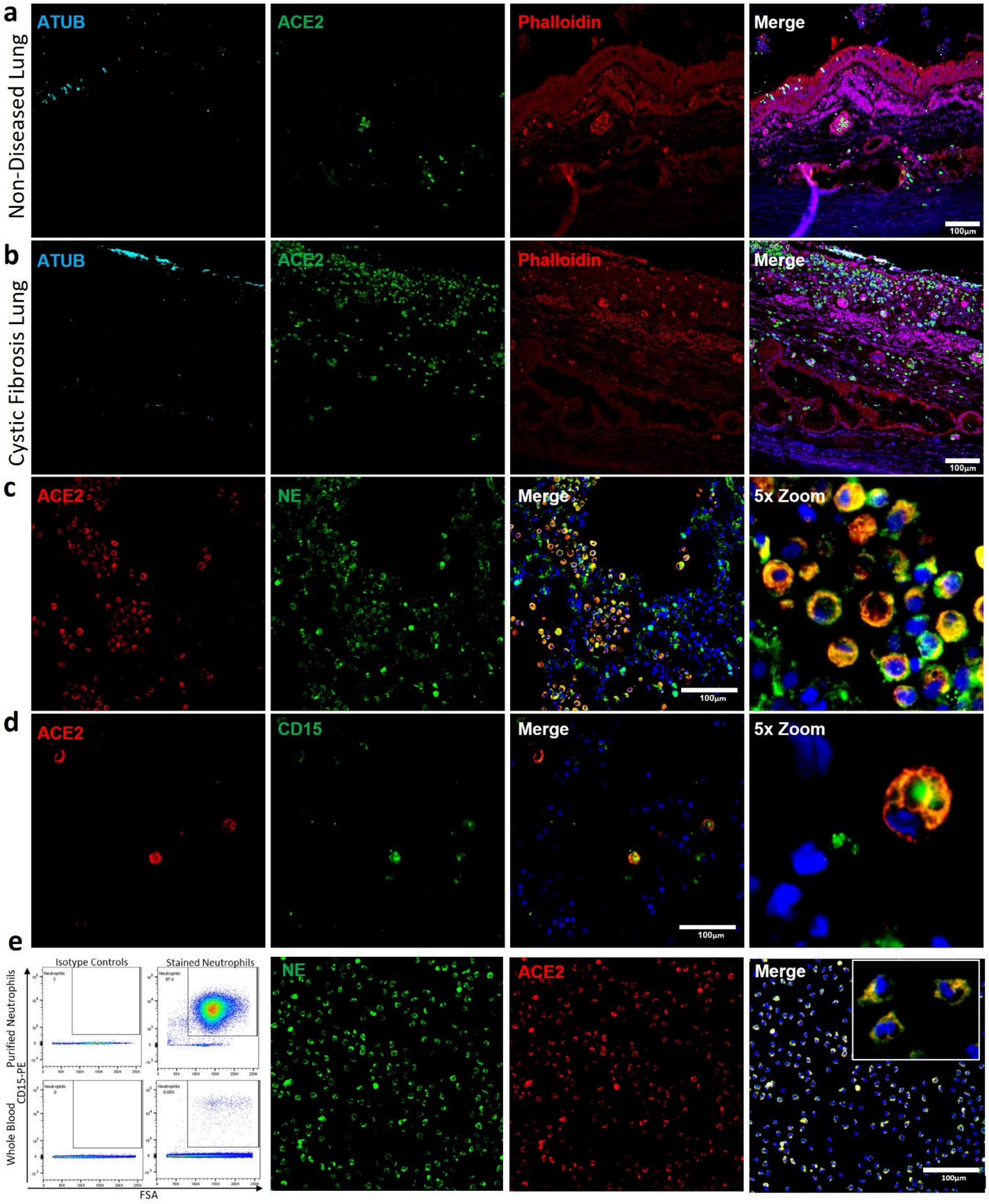

Supplemental figure S5, related to figure 5. Evidence for ACE2 expression in respiratory infiltrating neutrophils. A-b) Representative IF images of explanted human lung tissue from no underlying respiratory disease (a) or CF patients (b); for acetylated α-tubulin (cyan), ACE2 (green) and Phalloidin (red). Images highlight; increased ACE2+ Leukocyte infiltrating cystic fibrosis tissue; (c-d) Representative IF images of cystic fibrosis tissue for ACE 2 (red) NE (c) or CD15 (d) (green) demonstrating colocalization of neutrophil markers in

cells expressing ACE2. (e) FACS plot and subsequent IF staining of CD15+ Peripheral polymorphonuclear leukocytes isolated from human blood, showing expression of ACE2 in neutrophils. All IF images have nuclei counterstained with DAPI (blue) and scale bars represent 100 µm. All images are representative of 3 independent regions per donor at least 2 independent donors.

References

1. Carraro G, Langerman J, Sabri S, Lorenzana Z, Purkayastha A, Zhang G, Konda B, Aros CJ, Calvert BA, Szymaniak A, Wilson E, Mulligan M, Bhatt P, Lu J, Vijayaraj P, Yao C, Shia DW, Lund AJ, Israely E, Rickabaugh TM, Ernst J, Mense M, Randell SH, Vladar EK, Ryan AL, Plath K, Mahoney JE, Stripp BR, Gomperts BN. Transcriptional analysis of cystic fibrosis airways at single-cell resolution reveals altered epithelial cell states and composition. *Nat Med* 2021: 27(5): 806-814.
2. Butler A, Hoffman P, Smibert P, Papalexi E, Satija R. Integrating single-cell transcriptomic data across different conditions, technologies, and species. *Nat Biotechnol* 2018: 36(5): 411-420.
3. Hafemeister C, Satija R. Normalization and variance stabilization of single-cell RNA-seq data using regularized negative binomial regression. *Genome Biol* 2019: 20(1): 296.
4. Zappia L, Oshlack A. Clustering trees: a visualization for evaluating clusterings at multiple resolutions. *Gigascience* 2018: 7(7).
